# Venous Endothelial Cells Promote Osteoblast Differentiation More Effectively Than Arterial Cells via TGF‐β/BMP9 and Notch Pathway‐Related Gene Expression

**DOI:** 10.1002/cbf.70160

**Published:** 2026-01-16

**Authors:** Célio J. C. Fernandes, Rodrigo A. Foganholi da Silva, Marcel R. Ferreira, Willian F. Zambuzzi

**Affiliations:** ^1^ Bioassays and Cell Dynamics Lab, Department of Chemistry and Biochemistry, Bioscience Institute UNESP: Universidade Estadual Paulista Botucatu Sao Paulo Brazil; ^2^ School of Dentistry University of Taubaté Taubaté São Paulo Brazil; ^3^ CEEpiRG ‐ Center for Epigenetic Study and Genic Regulation, Program in Environmental and Experimental Pathology Paulista University São Paulo São Paulo Brazil; ^4^ Molecular Genetics and Bioinformatics Laboratory, Experimental Research Unit ‐ Unipex, School of Medicine São Paulo State University ‐ Unesp Botucatu São Paulo Brazil

**Keywords:** bone regeneration, endothelial cells, HUVEC, osteogenesis, signal transduction

## Abstract

The coupling between angiogenesis and osteogenesis is a key determinant of skeletal homeostasis, yet the influence of endothelial cell origin on osteoblast differentiation remains underexplored. Here, we investigated how venous (HUVECs) and arterial (HCAECs) endothelial cells differentially modulate the osteogenic phenotype of human osteoblasts via paracrine signaling. To better address this issue, the conditioned media (CM) from HUVECs significantly enhanced osteoblast differentiation, as evidenced by increased alkaline phosphatase activity, upregulation of canonical markers such as *Runx2*, *Osterix*, and *Osteocalcin*, and activation of matrix mineralization genes (*Tnap*, *Bsp*, *Col1a1*). In contrast, CM from HCAECs induced a markedly weaker response. qPCR analysis revealed that HUVEC‐CM robustly stimulated key osteoinductive pathways, including TGF‐β/BMP9 and Notch, with pronounced activation of SMADs, *Jagged*, and *Notch* receptors. Moreover, HUVEC‐CM promoted cytoskeletal remodeling via increased expression of *Integrin‐β1*, *FAK*, *Src*, and *Cofilin*, and favored ECM organization by repressing MMP activity and enhancing *Reck* expression. Hypoxia‐associated markers (*Hif1α*, *Vegf*) were also elevated in HUVEC‐treated osteoblasts, supporting enhanced angiogenic‐osteogenic coupling. Principal component and network analyses confirmed a distinct molecular clustering for HUVEC‐responsive genes. Altogether, our data demonstrate that venous endothelial cells, through their specific secretome, provide a more adequate microenvironment for osteoblast differentiation and mineralization compared to their arterial equivalent. These findings underscore the functional relevance of endothelial plasticity in bone regeneration and support the use of venous‐derived endothelial factors in bone tissue engineering strategies.

## Summary

- Bone regeneration depends on precise coupling between angiogenesis and osteogenesis, yet how endothelial origin shapes osteoblast fate is unclear.
- Here we show that venous endothelial cells (HUVECs) secrete factors that outperform arterial cells (HCAECs) in driving osteoblast differentiation and matrix mineralization, via coordinated activation of TGF‐β/BMP9–SMAD and Notch axes, reinforcement of focal‐adhesion/cytoskeletal programs, and induction of a hypoxia–VEGF feedback.
- These data identify a venous‐biased paracrine signature that aligns with bone microvascular biology and offers a clear criterion for selecting endothelial sources or secretomes to enhance bone tissue engineering and repair.

## 1 Introduction

Despite its static appearance, bone tissue has a remarkable capacity for remodeling, sustained by the orchestrated activity of osteoblasts and osteoclasts and tightly regulated by signaling molecules released by osteocytes, which constitute approximately 95% of all bone cells. Additionally, trophic factors secreted by other tissues, along with nutrients supplied via vascularization, are essential for maintaining bone health [1]. The relationship between bone remodeling and vascular cells is highly interconnected from the origin of both tissues, osteogenesis and angiogenesis, where each process, though distinct, contributes reciprocally to the establishment and maintenance of bone structure [2].

The formation of new blood vessels in the bone environment is a highly complex process involving the release of diverse signaling molecules. In turn, these vessels supply oxygen, nutrients, and growth factors required for bone cell function. Vascular‐derived factors, such as bone morphogenetic proteins (BMPs) and transforming growth factor‐beta (TGF‐β), play pivotal roles in recruiting mesenchymal stem cells and promoting their differentiation into osteoblasts, thereby facilitating new bone formation [3]. Moreover, blood vessels are responsible for the clearance of metabolic by‐products generated during bone remodeling. Conversely, osteoblasts release signaling molecules such as vascular endothelial growth factor (VEGF), platelet‐derived growth factor (PDGF), and fibroblast growth factor (FGF), which contribute to vascular growth and maintenance, reinforcing the angiogenic component of bone physiology [4].

In this context, extracellular matrix (ECM) remodeling is essential for sustaining bone homeostasis. This process is regulated by key proteins and enzymes such as bone sialoprotein (BSP), tissue non‐specific alkaline phosphatase (TNAP), and osteocalcin the latter increasingly recognized for its role in energy metabolism. These molecules act in a sequential and coordinated manner to promote mineral deposition and the formation of an organized bone matrix [5]. Importantly, hypoxia (a condition frequently observed in the bone microenvironment due to the high oxygen demand of metabolically active cells) is a critical regulator of both angiogenesis and osteogenesis [6] Under hypoxic conditions, hypoxia‐inducible factor 1‐alpha (HIF‐1α) becomes stabilized, leading to increased VEGF expression and the stimulation of neovascularization, thereby supporting bone tissue maintenance [7]. More recently, we developed vascular organoids in vitro and observed that vascular smooth muscle cells (VSMCs) contribute to hypoxia generation, thereby promoting osteogenic differentiation [8]. Furthermore, these VSMCs facilitate the osteoblast‐to‐osteocyte transition through the release of functional exosomes containing proteins and mRNAs [6, 8].

Endothelial cells lining blood vessels play a central role in angiogenesis. However, they are not phenotypically or functionally homogeneous. Arterial and venous endothelial cells exhibit distinct molecular signatures and biological behaviors, reflective of their specific roles in the circulatory system. These differences include their response to mechanical forces such as shear stress, and their potential to engage in paracrine signaling loops with surrounding tissues, a hypothesis that remains to be fully elucidated. Arterial endothelial cells are typically exposed to higher shear stress and are specialized in conducting oxygenated blood, whereas venous endothelial cells function under lower pressure and are responsible for returning deoxygenated blood to the heart [9]. Notably, Ramasamy et al. (2016) suggested that blood flow and endothelial Notch signaling are critical modulators of skeletal aging [10].

Emerging evidence suggests that the osteoinductive capacity of endothelial cells significantly affects their interaction with osteoblasts and, consequently, influences bone formation and remodeling [10]. The differential plasticity between arterial and venous endothelial cells may play a key role in directing osteoblastic differentiation and modulating the angiogenic‐osteogenic interface [11]. Therefore, the present study aims to explore this differential plasticity, specifically assessing how venous and arterial endothelial cells may influence the osteogenic phenotype through the release of trophic factors. Understanding these cell‐specific effects may pave the way for innovative therapeutic strategies to enhance bone regeneration. Moreover, this investigation contributes to the broader understanding of the interrelationship between angiogenesis and osteogenesis, offering new perspectives on the intricate mechanisms underlying healthy bone remodeling.

## 2 Materials and Methods

### 2.1 Cell Cultures

All cells were maintained at 37°C in a humidified atmosphere of 5% CO₂, with daily inspection of morphology and medium color. Osteoblastic cells: Primary human osteoblasts (HOEL; Homo sapiens, bone tissue) were isolated at HC‐FMB/UNESP and banked at LaBIO/UNESP under institutional ethics approval (CEP/CONEP–Brazil; CAAE: 81605024.4.0000.5411). Cells were expanded in low‐glucose DMEM supplemented with 10% FBS (Nutricell, Campinas, SP, Brazil) and antibiotics (penicillin 100 U/mL; streptomycin 100 µg/mL). Endothelial cells: Human coronary artery endothelial cells (HCAEC; Lonza, cat. CC‐2585) and primary human umbilical vein endothelial cells (HUVEC; pooled donors; Lonza, cat. CC‐2519) were maintained in EBM‐2 supplemented with the EGM‐2 SingleQuots kit, according to the manufacturer's instructions. Authentication and contamination control: Endothelial cell identities were verified by supplier Certificates of Analysis (Lonza), and HOEL identity by morphology and osteoblastic marker expression during expansion. All cultures were routinely screened and confirmed mycoplasma‐free (PCR/MycoAlert) prior to experiments.

### 2.2 Shear‐Stress and Collection of Conditioned Medium

Cells were cultured in appropriate medium until they reached semi‐confluence (90%) in modified Petri dishes, which were sterilized under UV light for 15 min. The medium was then replaced with DMEM supplemented with 1% antibiotic and 10% FBS. The semi‐confluent layers were subjected to Shear‐stress using an orbital shaker for 72 h [8, 12, 13]. The rotation was determined using the maximum Shear‐stress formula: τmax = a√ρη(2πf)^^3^, ensuring the rotation frequency was within the range of physiological arterial Shear‐stress (6–40 dynes/cm²), thus mimicking the mechanical forces encountered by blood flow. After 72 h, the conditioned medium was collected, centrifuged, and stored at −80°C for subsequent analysis. As a control, conditioned medium from cells maintained in static conditions (without rotation ‐ Static) was used, under the same culture conditions. Specifically, human umbilical vein endothelial cells (HUVECs) are referred to as the static HUVEC group, while those subjected to shear stress are indicated as d‐HUVECs. Similarly, human coronary artery endothelial cells (HCAECs) were identified as the static HCAEC group or d‐HCAECs, according to the experimental conditions.

### 2.3 Osteogenic Induction

As a positive control, we used a differentiation methodology from the literature, where β‐Glycerophosphate (10 mM), Dexamethasone (0.03 g/mL), and Ascorbic Acid (50 μg/mL) (Sigma‐Aldrich) were added. The cells were maintained in an incubator for 28 days, with the medium for each group being replaced every 3 days to maintain the biological properties of the study.

### 2.4 Cell Differentiation Analysis ‐ Alkaline Phosphatase (ALP)

Cells were plated at a density of 8,10^4^ cells/mL in 24‐well plates and, upon reaching semi‐confluence, treated with the different media as previously described, respecting the different evaluated groups. Cell differentiation was measured by the activity of the enzyme Alkaline Phosphatase (ALP), evaluated here using a staining method. The staining solution was prepared using the Sigma‐Aldrich kit (SIGMAFAST BCIP/NBT). It is important to note that this methodology was performed according to the manufacturer's instructions.

### 2.5 In Vitro Mineralization Analysis ‐ Alizarin RedS

Cells were plated and maintained exactly as detailed above. After 28 days of treatment, in vitro mineralization was measured by the ability of calcium incorporation to the matrix synthesized by osteoblasts through Alizarina RedS labeling. The culture medium was gently sucked and washed with PBS. Then the PBS was sucked out and 0.5 mL of 10% Formalin was added for cell attachment. The plate was held in that solution for 30 min at room temperature. After this period Formalin was sucked and Alizarin solution added (1%) and the plate incubated in the dark for 45 min. After this time, the wells were washed with PBS for five times. After the last wash, 0.5 mL of PBS was added to each well and the plate was photographed under an inverted microscope (Zeiss, Germany).

### 2.6 Zymography

The conditioned culture medium was centrifuged at 14,000 rpm for 15 min, and the protein concentration was determined using the Lowry method (Lowry et al. 1951). The gelatinolytic activity of the samples was assessed by fractionating the matrix metalloproteinases (MMP‐2 and MMP‐9) on a 12% polyacrylamide gel containing 4% gelatin. After fractionation, the MMPs were renatured in an aqueous solution of Triton X‐100 (2% w/v), incubated for 18 h in proteolysis buffer (Tris‐CaCl_2_) at 37°C, and stained with Coomassie Blue R‐250 0.05% staining solution for 3 h [14].

### 2.7 qPCR

After 28 days of treatment, TRIzol (500 μL) was added to collect samples for qPCR. RNA samples were quantitated using a plate reader (SYNERGY‐HTX multi‐mode reader, Biotek, USA). For cDNA synthesis, the High‐Capacity cDNA Reverse Transcription Kit from Applied Biosystems was used (2.0 μL of 10× RT Buffer, 0.8 μL 25× dNTP Mix (100 mM), 2.0 μL 10× RT Random Primers, 1.0 μL MultiScribe Reverse Transcriptase, and 4.2 μL Nuclease‐Free H_2_O). After this process, the samples were collected and stored in −80°C freezer. Reactions condition for all genes: 95°C − 15 s; 60°C − 30 s; 72°C − 30 s (Table 1).

**Table 1:** Expression primers sequences.

Gene	Primer	5′–3′ Sequence
*Bmpr1*	Forward	CATATTAGGCGTGTGCCAAAAA
	Reverse	GCTTGTGCTTGCTGTCGTTC
*Smad2*	Forward	GCCGCCAGTTGTGAAGAGAC
	Reverse	TGGAGACGACCATCAAGAGACC
*Smad3*	Forward	GCTGACACGGAGACACATCG
	Reverse	AGCCTCAAAGCCCTGGTTG
*Smad4*	Forward	CTTTGAGGGACAGCCATCGT
	Reverse	GCCACAGAAATGTTGGGAAA
*Smad5*	Forward	CGGCCGAGCTGCTAATAAAG
	Reverse	TTCATTGGGTCAAGTCTCGC
*Smad6*	Forward	TACTCTCGGCTGTCTCCTC
	Reverse	GAGTTGGTAGCCTCCGTTTC
*Smad7*	Forward	GCTGAAACAGGGGGAACGA
	Reverse	AGTATGCCACCACGCACCA
*Tgf‐β1*	Forward	CAACGAAATCTATGACAAGTTCAAGCAG
	Reverse	CTTCTCGGAGCTCTGATGTG
*Runx2*	Forward	CCGTCCATCCACTCTACCAC
	Reverse	ATGAAATGCTTGGGAACTGC
*Osterix*	Forward	CCAGGCAACACTCCTACTCC
	Reverse	GCCTTGCCATACACCTTGC
*Ptch*	Forward	GGGTCCTCGCTTACAAACTC
	Reverse	ATGATGCCATCTGCGTCTAC
*Sufu*	Forward	GAGGGCGAAGTAATTTGTGG
	Reverse	AGCCCTCCTTCTGAGTGCTT
*Shh*	Forward	CACCGAGCAGTGGATATGTG
	Reverse	AGTGGCCAGGAGTGAAACTG
*Tnap*	Forward	TTTATAAGGCGGCGGGGGTG
	Reverse	AGCCCAGAGATGCAATCGAC
*Npp1*	Forward	TTTGCCGATTGAGGATTTTC
	Reverse	CCACTGACGACATTGACACC
*Vegf*	Forward	TGCAGATTATGCGGATCAAACC
	Reverse	TGCATTCACATTTGTTGTGCTGTAG
*Vegf‐r*	Forward	CAGGCCCAGTTTCTGCCATT
	Reverse	TTCCAGCTCAGCGTGGTCGTA
*Notch1*	Forward	GGAAGTGTGAAGCGGCCAATG
	Reverse	ATAGTCTGCCACGCCTCTGC
*Notch2*	Forward	TGTCGAGATGGCTATGAACCCTG
	Reverse	GCAGCGGTTCTTCTCACAGG
*Nocth3*	Forward	TGTCTGCCAGAGTTCAGTGGTG
	Reverse	AGGAGCAGAGGAAGCGTCCATC
*Notch4*	Forward	TTGTCCTCCCTCCTTCTGTTCC
	Reverse	AGAAGTCCCGAAGCTGGCAC
*Jag1*	Forward	TGCCTCTGTGAGACCAACTG
	Reverse	GTTGGGTCCTGAATACCCCT
*Jag2*	Forward	GTGGCAAGAACTGCTCCGTG
	Reverse	TGCCTCTGTGAGACCAACTG
*Bmp9*	Forward	AGAACGTGAAGGTGGATTTCC
	Reverse	CGCACAATGTTGG ACGCTG
*Hif‐1α*	Forward	CATAAAGTCTGCAACATGGAAGGT
	Reverse	ATTTGATGGGTGAGGAATGGGTT
*Hif‐1β*	Forward	CAAGCCCCTTGAGAAGTCAG
	Reverse	GAGGGGCTAGGCCACTATTC
*Integrin‐β1*	Forward	GCCGCGCGGAAAAGATGAA
	Reverse	TGCTGTTCCTTTGCTACGGT
*Src*	Forward	CAACACAGAGGGAGACTGGT
	Reverse	AGCTTCTTCATGACCTGGGC
*Fak*	Forward	TCAGCTCAGCACAATCCTGG
	Reverse	CTGAAGCTTGACACCCTCGT
*Cofilin*	Forward	TGTGCGGCTCCTACTAAACG
	Reverse	TCCTTGACCTCCTCGTAGCA
*Mmp2*	Forward	AGCTCCCGGAAAAGATTGATG
	Reverse	CAGGGTGCTGGCTGAGTAGAT
*Mmp9*	Forward	CACGCACGACGTCTTCCA
	Reverse	AAGCGGTCCTGGCAGAAAT
*Timp1*	Forward	CCGCAGCGAGGAGTTTCTC
	Reverse	GAGCTAAGCTCAGGCTGTTCCA
*Timp2*	Forward	CGACATTTATGGCAACCCTATCA
	Reverse	GGGCCGTGTAGATAAACTCTATATCC
*Reck*	Forward	TGCAAGCAGGCATCTTCAAA
	Reverse	ACCGAGCCCATTTCATTTCTG
*Col1a1*	Forward	AACCAAGGCTGCAACCTGGA
	Reverse	GGCTGAGTAGGGTACACGCAGG
*Alp*	Forward	CGGGCACCATGAAGGAAA
	Reverse	GGCCAGACCAAAGATAGAGTT
*Bsp*	Forward	GTTGCGTCTTGGAAGTGAGA
	Reverse	CAGCTGGCTGATCACTCAAA
*Osteocalcin*	Forward	ATGAGAGCCCTCACACTCCTC
	Reverse	GCCGTAGAAGCGCCGATAGGC
*Gapdh*	Forward	GACTCATGACCACAGTCCATGC
	Reverse	AGAGGCAGGGATGATGTTCTG

### 2.8 Bioinformatic Analysis

The gene expression values for all experimental groups underwent principal component analysis (PCA) in the R version 4.1.3. This involved using the base, stats, and factoextra packages, and ensuring that the values were centralized and scaled beforehand. Furthermore, a gene correlation network analysis was conducted using the corrr package, based on relative gene expression values, with the correlation threshold set at R^2 > 0.75. Subsequently, Cytoscape was employed to visualize and analyze the network, while the MCODE app was utilized to carry out cluster analysis.

### 2.9 Statistical

The results were represented as mean ± standard deviation (SD). Statistical significance was determined using one‐way ANOVA (non‐parametric) with Tukey's post‐test to compare all pairs of groups, with *p* < 0.05 considered statistically significant and *p* < 0.0001 considered highly significant. GraphPad Prism 9 software was used for analysis. For particle counting, Image‐J software was used to determine the number of points in the registered photo area, and the groups were standardized by the percentage of the control for statistical analysis. For determining the number of star‐shaped cells, the number of nuclei in the registered photo area was counted, followed by statistical analysis.

## 3 Results

### 3.1 Venous Endothelial Cells Promote Osteoblastic Differentiation and Matrix Mineralization More Effectively Than Arterial Cells

**Figure 1:**
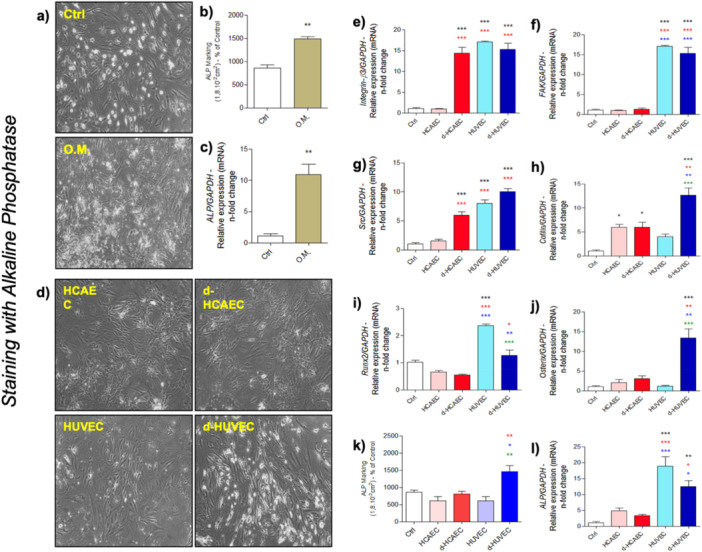
HUVEC‐conditioned medium modulates osteoblast differentiation markers and matrix mineralization. After 72 h under Shear‐stress or static conditions, endothelial cell‐conditioned medium (HUVEC and HCAEC) was collected to treat osteoblasts for 28 days. At the end, cells were evaluated for Alkaline Phosphatase activity profile (a‐d, k). Additionally, cells were collected in TRIzol for RNA extraction and investigation of gene expression involved in cell adhesion: (e) *Integrin*, (f) *Fak*, (g) *Src*, and (h) *Cofilin*. Other markers involved in osteoblast differentiation genotype were assessed: (i) *Runx2*, (j) *Osterix*, and (l) *Alp*. As a positive control, Osteogenic Medium (O.M.) was used. GAPDH was used as a reference gene for expression normalization. *p < 0.0292; **p < 0.0094; ***p < 0.0010: Statistical difference compared to the control group. 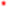 *p* < 0.0292; 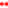 *p* < 0.0094; 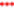 *p* < 0.0010: Statistical difference compared to the HCAEC group. 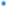 *p* < 0.0292; 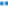 *p* < 0.0094; 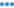 *p* < 0.0010: Statistical difference compared to the d‐HCAEC group. 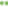 *p* < 0.0094; 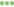 *p* < 0.0010: Statistical difference compared to the HUVEC group.

After 72 h of conditioning under either static or shear‐stress conditions, the culture media from venous (HUVECs) and arterial (HCAECs) endothelial cells were collected and used to treat human osteoblasts over a 28‐day period. This setup allowed us to evaluate the influence of endothelial‐derived trophic signals on osteoblast behavior throughout the differentiation and mineralization process. A consistent finding across the experiments was the higher osteoinductive potential of HUVEC‐conditioned media. Osteoblasts exposed to these media exhibited markedly increased activity of alkaline phosphatase (ALP) and significantly higher expression of canonical osteogenic transcription factors, including *Runx2* and *Osterix*, compared to those treated with HCAEC‐conditioned media (Figure 1a–j,l). These effects were observed regardless of whether the HUVECs had been subjected to shear stress, suggesting a baseline secretory phenotype more favorable to osteogenic commitment.

In addition to transcriptional markers, the expression of genes related to cytoskeletal organization and focal adhesion, *Integrin‐β1*, *FAK*, *Src*, and *Cofilin*, was clearly elevated in the HUVEC groups (Figure 1e–h), implying a more dynamic interface between the osteoblasts and their surrounding matrix. These results suggest that venous endothelial cells enhance not only differentiation but also the mechanical readiness of osteoblasts to engage with and remodel their extracellular environment.

### 3.2 Venous Endothelial‐Derived Factors Enhance Osteogenic Signaling and Matrix Mineralization

**Figure 2:**
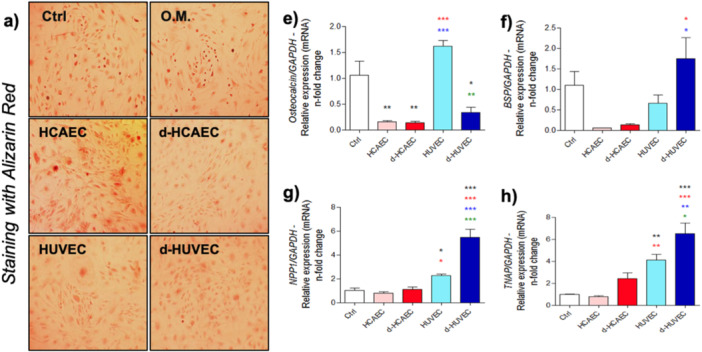
Effect of endothelial cell‐conditioned medium on extracellular matrix regulation markers in osteoblasts**.** Endothelial cells from artery (HCAEC) and vein (HUVEC) were used to condition the culture medium under two conditions: Shear‐stress and Static. After 72 h, the medium was collected and used to treat osteoblasts. At the end of 28 days, Alizarin Red staining was performed to assess matrix mineralization (a). The expression of *Osteocalcin* (b), *Bone Sialoprotein* – *Bsp* (c), *Npp1* (d), and *Tnap* (e) was also evaluated. *Gapdh* was considered an endogenous gene for normalizing ΔΔCT values. *p < 0.0292; **p < 0.0094; ***p < 0.0010: Statistical difference compared to the control group. 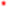 *p* < 0.0292; 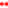 *p* < 0.0094; 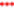 *p* < 0.0010: Statistical difference compared to the HCAEC group. 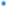 *p* < 0.0292; 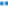 *p* < 0.0094; 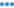 *p* < 0.0010: Statistical difference compared to the d‐HCAEC group. 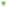 *p* < 0.0292; 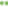 *p* < 0.0094; 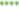 *p* < 0.0010: Statistical difference compared to the HUVEC group.

To further assess the osteoinductive capacity of endothelial‐derived signals, we performed Alizarin Red staining to evaluate matrix mineralization (Figure 2a). We then examined genes associated with calcium and phosphate homeostasis, as well as key regulatory pathways involved in osteoblast differentiation. Osteoblasts treated with HUVEC‐conditioned media showed a marked increase in Osteocalcin expression, a late marker of maturation, while HCAEC‐treated groups exhibited little or no induction, and in some cases, downregulation (Figure 2b). A similar expression pattern was observed for *Bsp, Tnap*, and *Npp1*, all of which play essential roles in matrix mineral deposition (Figure 2c–e). These findings indicate that venous endothelial factors more effectively promote the transcriptional program necessary for mineralization.

**Figure 3:**
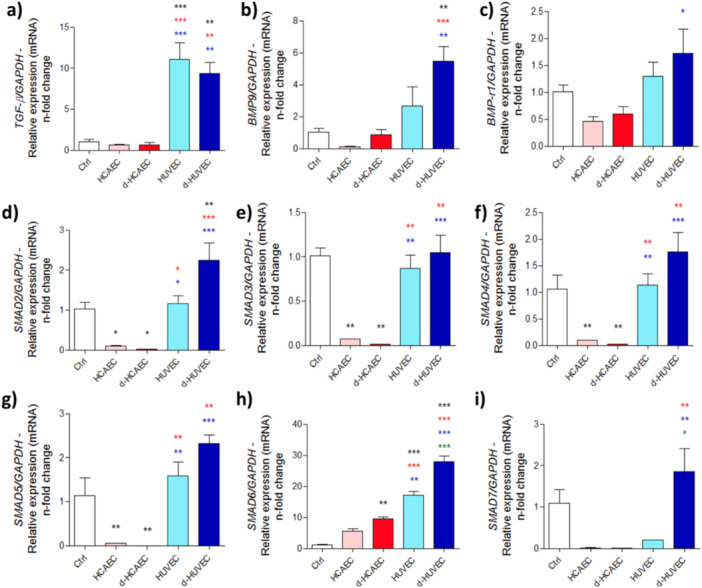
HUVEC activates the expression of genes involved in the TGF‐β pathway in osteoblasts. Osteoblasts received conditioned medium from HCAEC and HUVEC for 28 days, and at the end, they were collected for RNA extraction, cDNA synthesis, and gene expression analysis. We investigated the expression profile of *Tgf‐β* (a), *Bmp9* (b), *Bmp receptor* (c), and *Smad* group acting as transcription factors (d–i). Experiments represent *n* = 3. *Gapdh* was used as an internal expression marker. *p < 0.0292; **p < 0.0094; ***p < 0.0010: Statistical difference compared to the control group. 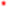 *p* < 0.0292; 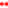 *p* < 0.0094; 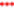 *p* < 0.0010: Statistical difference compared to the HCAEC group. 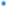 *p* < 0.0292; 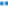 *p* < 0.0094; 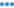 *p* < 0.0010: Statistical difference compared to the d‐HCAEC group. 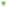 *p* < 0.0292; 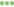 *p* < 0.0010: Statistical difference compared to the HUVEC group.

Complementing these results, gene expression analysis revealed that HUVEC‐conditioned media robustly activated osteogenic signaling pathways, particularly *Tgf‐β1* and *Bmp9*, along with several members of the SMAD family (*Smad2, Smad3, Smad4, Smad5*, and *Smad7*) (Figure 3a–i). In contrast, the response to HCAEC‐derived media was significantly weaker across all these genes. Taken together, these data suggest that venous endothelial cells possess a distinct and more potent secretory profile capable of triggering molecular cascades critical for osteoblast lineage commitment and functional maturation.

### 3.3 Venous Endothelial Signals Favor Extracellular Matrix Preservation and Promote Angiogenic Feedback in Osteoblasts

**Figure 4:**
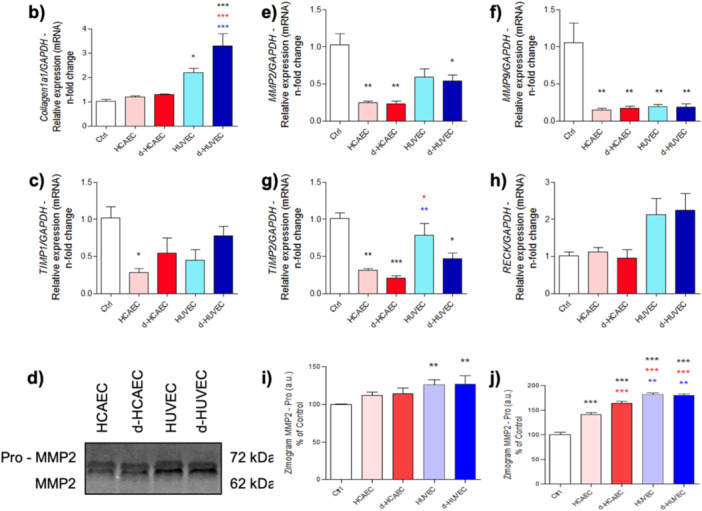
Expression of genes involved in extracellular matrix remodeling. Osteoblasts treated with conditioned medium from HUVEC and HCAEC were collected in TRIzol after 28 days of treatment. The expression of *Collagen 1a1*, *Mmp2*, *Mmp9*, *Timp1*, *Timp2*, and *Reck* was evaluated (a–f). Additionally, the medium was collected to investigate the activity of Metalloproteinases potentially released in the Osteoblasts' medium (g–i). *p < 0.0292; **p < 0.0094; ***p < 0.0010: Statistical difference compared to the control group. 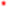 *p* < 0.0292; 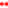 *p* < 0.0094; 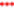 *p* < 0.0010: Statistical difference compared to the HCAEC group. 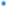 *p* < 0.0292; 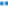 *p* < 0.0094; 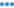 *p* < 0.0010: Statistical difference compared to the d‐HCAEC group. 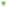 *p* < 0.0292; 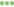 *p* < 0.0010: Statistical difference compared to the HUVEC group.

Analysis of extracellular matrix (ECM) remodeling revealed that osteoblasts treated with HUVEC‐conditioned media exhibited significantly elevated expression of *Col1a1*, consistent with the mineralization‐enhancing effects previously observed (Figure 4a). In parallel, transcription of the matrix metalloproteinases *Mmp2* and *Mmp9* was markedly reduced in these groups (Figure 4b–c), suggesting a lower matrix turnover rate. Despite this transcriptional repression, gelatinolytic activity of MMPs remained moderate across all groups (Figure 4g–i). The expression of MMP inhibitors *Timp1* and *Timp2* was generally decreased regardless of treatment, but only the HUVEC groups showed a tendency toward upregulation of *Reck* (Figure 4f), a key regulator of ECM integrity. These results suggest that venous endothelial signals contribute to a remodeling profile characterized by matrix preservation and structural maintenance.

**Figure 5:**
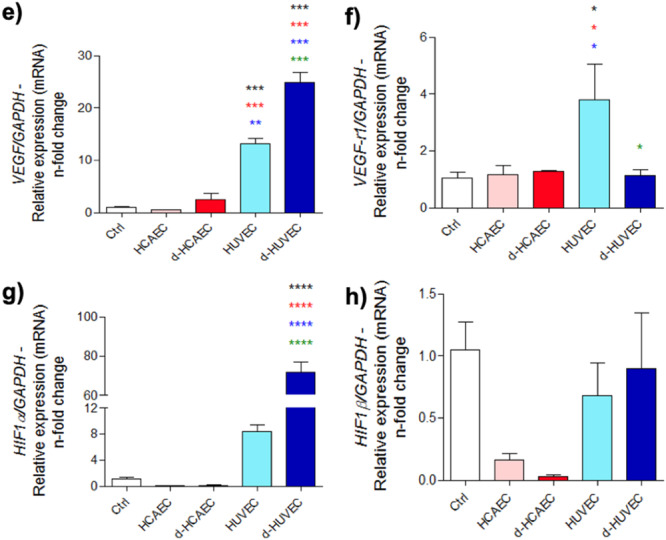
Induction of VEGF and HIF expression in endothelial cell‐challenged osteoblasts. Osteoblasts were treated for 28 days with conditioned medium from endothelial cells; the medium was changed every 3 days. Gene expression was evaluated using real‐time PCR, and expression values were normalized by ΔΔCT. We investigated genes: (a) *Vegf*, (b) *Vegf‐r1*, (c) *Hif‐1α*, and (d) *Hif‐1β*. *p < 0.0292 ***p < 0.0010; ****p < 0.0001: Statistical difference compared to the control group. 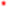 *p* < 0.0292; 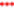 *p* < 0.0010; 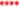 *p* < 0.0001: Statistical difference compared to the HCAEC group. 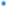 *p* < 0.0292; 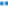 *p* < 0.0094; 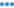 *p* < 0.0010; 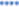 *p* < 0.0001: Statistical difference compared to the d‐HCAEC group. 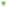 *p* < 0.0292; 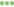 *p* < 0.0010; 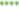 *p* < 0.0001: Statistical difference compared to the HUVEC group.

In addition to ECM‐related changes, HUVEC‐derived factors significantly increased the expression of hypoxia‐responsive and pro‐angiogenic genes in osteoblasts, including *Hif‐1α*, *Hif‐1β*, *Vegf*, and *Vegf‐r1* (Figure 5a–d). This indicates that venous endothelial cells not only promote osteogenic maturation but also stimulate a feedback loop that enhances the osteoblasts’ capacity to support angiogenesis. In contrast, HCAEC‐treated groups showed limited or no induction of these pathways, reinforcing the superior paracrine influence of venous endothelium in coordinating the vascular–bone interface.

### 3.4 Venous Endothelial Cells Sustain Morphogenetic Signaling via Hedgehog and Notch Pathways in Osteoblasts

**Figure 6:**
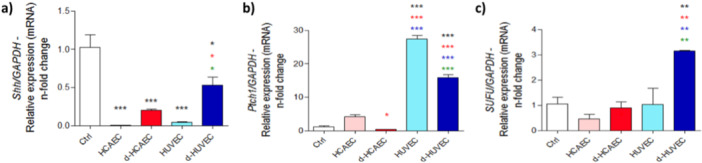
Effect of crosstalk between endothelial cells and osteoblasts on Sonic Hedgehog pathway expression. Osteoblasts were collected in TRIzol for RNA extraction after 28 days of treatment with conditioned medium from endothelial cells. cDNA was synthesized to assess the expression of Sonic Hedgehog (*Shh*), Patched (*Ptch1*), and *Sufu* (a–c). *Gapdh* was used as an endogenous gene to normalize ΔΔCT values. *p < 0.0292 **p < 0.0094; ***p < 0.0010: Statistical difference compared to the control group. 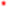 *p* < 0.0292; 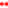 *p* < 0.0094; 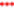 *p* < 0.0010: Statistical difference compared to the HCAEC group. 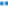 *p* < 0.0094; 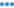 *p* < 0.0010: Statistical difference compared to the d‐HCAEC group. 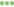 *p* < 0.0010: Statistical difference compared to the HUVEC group.

Although *Shh* expression was reduced across all treatment conditions (Figure 6a), HUVEC‐conditioned media induced selective upregulation of downstream transduction components of the Hedgehog pathway, notably *Ptch1* and *Sufu* (Figure 6b–c). These elements are essential for maintaining pathway activity and regulating GLI‐mediated transcription, which may explain the previously observed increase in *Vegf* expression. This indicates that HUVEC‐derived signals sustain Hedgehog pathway functionality in osteoblasts, even in the absence of elevated ligand levels, reinforcing their role in modulating angiogenesis‐related transcriptional programs.

**Figure 7:**
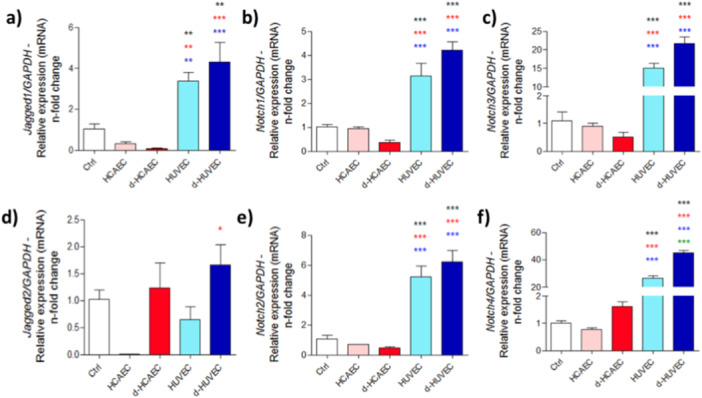
Notch pathway expression in osteoblasts challenged with trophic factors released by endothelial cells. Conditioned medium was used to treat osteoblasts, and after 28 days, RNA was collected. Real‐time PCR was used to evaluate the expression of *Jagged*1, *Jagged2*, *Notch1*, *Notch2*, *Notch3*, and *Notch4* (a–f). *Gapdh* was considered an endogenous gene to normalize ΔΔCT values. **p < 0.0094; ***p < 0.0010: Statistical difference compared to the control group. 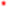 *p* < 0.0292; 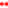 *p* < 0.0094; 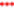 *p* < 0.0010: Statistical difference compared to the HCAEC group. 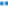 *p* < 0.0094; 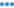 *p* < 0.0010: Statistical difference compared to the d‐HCAEC group. 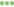 *p* < 0.0010: Statistical difference compared to the HUVEC group.

Complementing this, osteoblasts exposed to HUVEC‐conditioned media also exhibited a robust activation of the Notch signaling axis. Genes encoding Notch receptors (*Notch1–4*) and ligands (*Jagged1*, *Jagged2*) were consistently upregulated under both static and shear‐stressed HUVEC conditions, whereas expression in HCAEC‐treated cells remained minimal or inconsistent (Figure 7a–f). This highlights the unique ability of venous endothelial cells to engage key morphogenetic pathways that govern cell fate decisions and intercellular communication, thereby contributing to a microenvironment that supports coordinated osteogenic and angiogenic processes essential for bone tissue regeneration.

### 3.5 Principal Component and Correlation Network Analyses Reveal Huvec‐Specific Gene Expression Architecture

**Figure 8:**
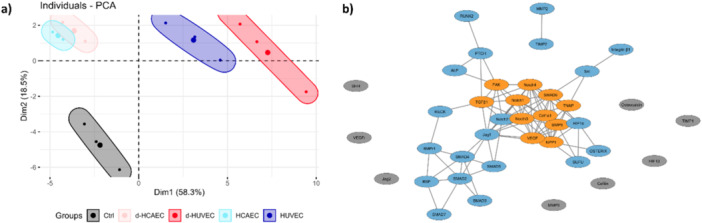
Bioinformatics analysis of gene expression. The relative gene expression values of each gene were centered, scaled, and then used as input for Principal Component Analysis. In Figure 8a, you can see the individual plot of the group in the two major dimensions. Subsequently, the pairwise correlation between each gene was utilized to construct a gene correlation network (depicted in Figure 8b), where eight genes have no interactions (gray nodes), and eleven genes form a cluster (orange nodes).

To gain a systems‐level understanding of the relationships within the gene expression dataset, we performed a principal component analysis (PCA). Each gene was considered a variable, and each replicate was treated as an individual observation. The scree plot (Figure S1a) displays the proportion of variance explained by the ten principal components generated. Dimensionality reduction revealed that the first principal component (Dim1), plotted along the x‐axis in Figure 8a, accounts for 58.3% of the total variance and clearly separates the HUVEC and d‐HUVEC groups from the HCAEC and d‐HCAEC groups, with the control group positioned between them. The second component (Dim2), representing 18.5% of the variance, further distinguishes the experimental groups, clustering all treated groups on the positive side of the *y* axis, while the control group remains in the negative quadrant. Figure S1b–d provide the contributions of individual genes to dimensions 1, 2, and 3, respectively, sorted in descending order.

To further explore co‐regulatory patterns, we constructed a gene correlation network based on pairwise Pearson correlations of relative gene expression values. A correlation threshold of *R*² > 0.75 was applied to define network edges (Figure 8b). Using the MCODE plugin, we identified a distinct gene cluster consisting of 11 highly interconnected nodes (orange), including *Bmp9*, *Col1a1*, *Fak*, *Notch1*, *Notch3*, *Notch4*, *Npp1*, *Tgf‐β1*, *Tnap*, and *Vegf*. In contrast, eight genes, *Shh*, *Vegf‐r*, *Jag2*, *Hif‐1β*, *Cofilin*, *Mmp9*, *Timp1*, and *Osteocalcin*, exhibited insufficient correlation to be integrated into the network structure and are represented as isolated gray nodes. *Timp2* and *Mmp2* showed a direct connection between themselves but remained disconnected from the main cluster. Figure S1e presents a bar plot illustrating the degree centrality of each gene, highlighting those with the highest number of connections within the network.

## 4 Discussion

Over the last years, the interaction between endothelial cells and osteoblasts has been centered as a pivotal role in skeletal development and regeneration, particularly in the coordination between bone formation and vascular function [1]. In this context, the use of conditioned medium from cell culture in vitro allows the establishment of a complex pool of trophic factors released by endothelial cells, collectively referred to as the secretome, which can influence osteoblastic phenotype. Our findings demonstrate that conditioned medium derived from venous endothelial cells (HUVECs) markedly enhances osteoblast differentiation and mineralization, a biological effect not replicated by arterial endothelial cells (HCAECs). This biological response is reflected in the sustained activation of key transcriptional regulators, including *Runx2*, *Osterix*, and *Alp*, as well as genes related to matrix organization, mineral homeostasis, and cell adhesion. It is important to note, however, that while the myriad of components of secretome was not completely elucidated, our results clearly demonstrate its functional impact on osteoblasts.

A central mechanism underlying this osteoinductive effect is the classical activation of the TGF‐β and BMP9 signaling axes, particularly through SMAD‐dependent transcriptional cascades, enhancing osteogenic outcomes. As shown by Wu et al. (2016), BMP9 is among the most potent osteogenic morphogens, requiring coordinated SMAD activation to regulate osteoblast differentiation [15]. Here, our data confirm that HUVEC‐conditioned medium significantly upregulates *Bmp9*, *Tgf‐β1*, and downstream *Smad2/3/4/5/7*, supporting a transcriptional environment conducive to bone matrix production and mineral deposition. Interestingly, SMAD6 was upregulated in d‐HCAEC, HUVEC, and d‐HUVEC, while SMAD7 increased only in d‐HUVEC, which may seem paradoxical given their canonical inhibitory roles. This context‐dependent upregulation likely reflects a feedback mechanism and selective modulation of specific TGF‐β/BMP signaling branches, allowing effective osteogenic promotion while maintaining regulatory control. These findings suggest that SMAD6/7 contribute to a balanced transcriptional environment, fine‐tuning osteoblast differentiation in response to endothelial paracrine cues.

Interestingly, this molecular profile is accompanied by increased expression of integrin‐related genes (*Integrin‐β1*, *FAK*, *Src*, *Cofilin*), implicating cytoskeletal remodeling and focal adhesion signaling in osteoblast maturation. As previously reported in FAK‐knockout mouse models [16], disruptions in integrin‐mediated signaling compromise matrix organization and repair, positioning the relevance of this pathway in bone homeostasis. The concurrent upregulation of these adhesion molecules in response to venous endothelial factors suggests that HUVECs support not only osteoblast differentiation but also their functional integration into the extracellular matrix, being a hallmark for osteoblast survival.

A further layer of complexity is introduced by the differential regulation of mineralization‐related genes, including *Osteocalcin*, *Bsp*, *Tnap*, and *Npp1*, all significantly upregulated in HUVEC‐treated groups. These genes are tightly regulated by phosphatase activity and extracellular nucleotide metabolism, known to modulate matrix mineral deposition [17, 18, 19, 20]. The simultaneous increase in ALP enzymatic activity reinforces the functional maturation of osteoblasts responding to HUVEC.

Notably, HUVECs also stimulated angiogenic feedback mechanisms, evidenced by the upregulation of *Vegf*, *Vegf‐r1*, *Hif‐1α*, and *Hif‐1β*. These findings are consistent with the role of hypoxia‐inducible signaling in coordinating vascular expansion with osteogenesis [6, 21, 22]. The paracrine induction of *Vegf* in osteoblasts may represent a positive feedback loop supporting type H capillary formation, as proposed by Ramasamy et al. (2016), which described the spatial and functional coupling between endothelial cells and osteoprogenitors in bone [23].

In contrast, HCAEC‐conditioned medium failed to elicit a comparable osteogenic or angiogenic response considering the same experimental conditions. This divergence likely reflects the intrinsic phenotypic specialization of arterial versus venous endothelial cells. Arterial endothelium, chronically exposed to high shear stress and pulsatile pressure, is adapted to maintain vascular tone and barrier integrity. Conversely, venous endothelium, derived from lower‐pressure environments, exhibits greater plasticity and secretory potential, particularly in contexts requiring tissue remodeling or regeneration. This interpretation aligns with our observation that HUVECs, but not HCAECs, activate the Notch pathway (*Notch1–4*, *Jagged1*, *Jagged2*), which plays a central role in coordinating osteogenesis and angiogenesis [24, 25, 26, 27, 28]. Notch activation in osteoblasts treated with HUVEC media reinforces the concept of functional specialization, suggesting that venous endothelial cells mimic or promote signals typical of regenerative type H vessels [1, 23, 29].

Furthermore, although *Shh* ligand expression was reduced in all experimental conditions, HUVEC‐treated osteoblasts displayed increased expression of *Ptch1* and *Sufu*, indicating downstream Hedgehog pathway activation. This suggests that even in the absence of ligand overexpression, HUVECs maintain morphogenetic signaling via non‐canonical regulation or residual paracrine factors, potentially linked to GLI‐mediated transcription of angiogenic genes such as *Vegf*.

Collectively, these findings reveal a distinct molecular signature driven by venous endothelial cells that promotes osteoblast differentiation, matrix maturation, and vascular–bone coupling. Importantly, the differences observed between HUVECs and HCAECs may stem from their physiological origin: venous endothelial cells operate under pressure conditions more similar to the bone [1, 23, 29] microenvironment, which is relatively low in perfusion pressure and rich in metabolic exchange. This hemodynamic compatibility may partially explain the superior osteoinductive profile of HUVECs, as bone capillaries (including type H vessels) share structural and functional traits with venous endothelium [23, 30].

**Figure 9:**
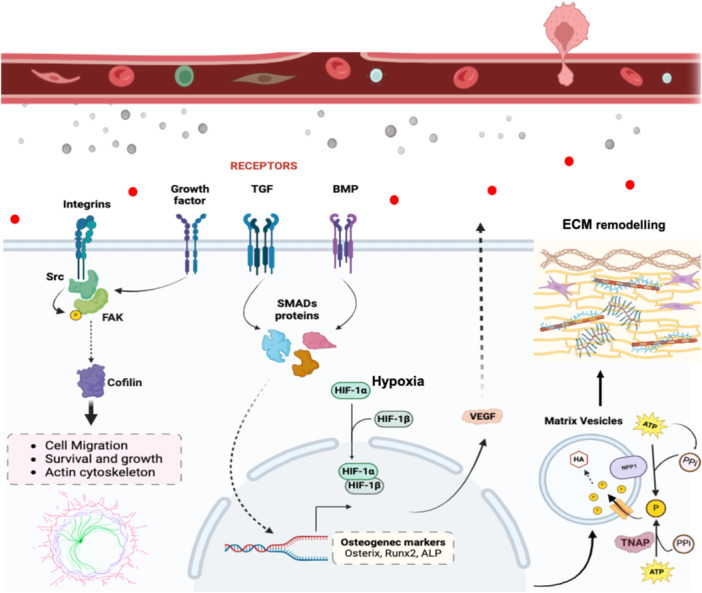
Molecular mechanisms involved in osteogenesis and ECM remodeling mediated by endothelial cell‐derived signals. This scheme assembly the key signaling pathways and cellular processes involved in osteoblast differentiation and extracellular matrix (ECM) remodeling as influenced by endothelial cell‐derived factors. Integrin's initiate cell migration, survival, and growth by activating Src and FAK, leading to cytoskeletal rearrangements via Cofilin. Growth factors and TGF‐β activate their respective receptors, triggering the SMADs protein complex that translocate to the nucleus, promoting the expression of osteogenic markers such as Osterix, Runx2, and ALP. BMP signaling further enhances this process through SMADs‐mediated transcriptional regulation. Hypoxia within the bone microenvironment stabilizes HIF‐1α and HIF‐1β, which together induce the expression of VEGF, supporting angiogenesis and osteogenesis. Matrix vesicles, containing enzymes such as TNAP and NPP1, play a critical role in ECM remodeling by regulating phosphate homeostasis and facilitating mineral deposition, crucial for bone matrix formation. This integrated signaling network underscores the coupling of angiogenesis and osteogenesis, highlighting the significant role of endothelial cells in bone regeneration.

In conclusion, our study provides compelling evidence that venous endothelial cells, through their secretome, are better suited than their arterial equivalents to promote osteoblast function and potentially bone regeneration (Figure 9). The activation of osteogenic, angiogenic, and morphogenetic pathways by HUVEC‐conditioned medium demonstrates its translational potential for regenerative medicine applications. However, it is important to note that the molecular composition of the secretome was not explored in detail in this study; future investigations using proteomic and metabolomic approaches will be critical to identify the specific factors responsible for the observed effects. These findings not only validate the use of HUVECs as a robust model for studying bone–vascular interactions in vitro, but also emphasize the need to account for endothelial heterogeneity and hemodynamic context when developing endothelial‐based strategies for skeletal repair. Importantly, the present results complement our previous findings, which demonstrated that vascular smooth muscle cells (VSMCs) actively promote the transition of osteoblasts into osteocytes [8]. Together, these observations reinforce the concept that blood vessels are not merely passive tissues for nutrient and oxygen delivery, but active participants in bone tissue morphogenesis and remodeling. Finally, the translational relevance of these results may be greatest in clinical contexts such as complex fractures, critical‐sized bone defects, and conditions of low bone density, including osteoporosis, where strategies modulating endothelial paracrine signaling could enhance bone regeneration. This evolving understanding of the vascular contribution to skeletal biology opens new avenues for therapeutic intervention in bone‐related pathologies.

## Supporting information

SUPP_DATA.

## Data Availability

The data that support the findings of this study are available on request from the corresponding author. The data are not publicly available due to privacy or ethical restrictions.
