# Supplementary material for: Venous Endothelial Cells Promote Osteoblast Differentiation More Effectively Than Arterial Cells via TGF‐β/BMP9 and Notch Pathway‐Related Gene Expression
Source: Cell Biochem Funct. 2026 Jan 16;44(1):e70160. doi: 10.1002/cbf.70160 (PMC12809378; doi:10.1002/cbf.70160)
Supplement: Supplementary file 1 — SUPP_DATA. [file CBF-44-e70160-s001.docx]

**SUPPLEMENTARY DATA:**


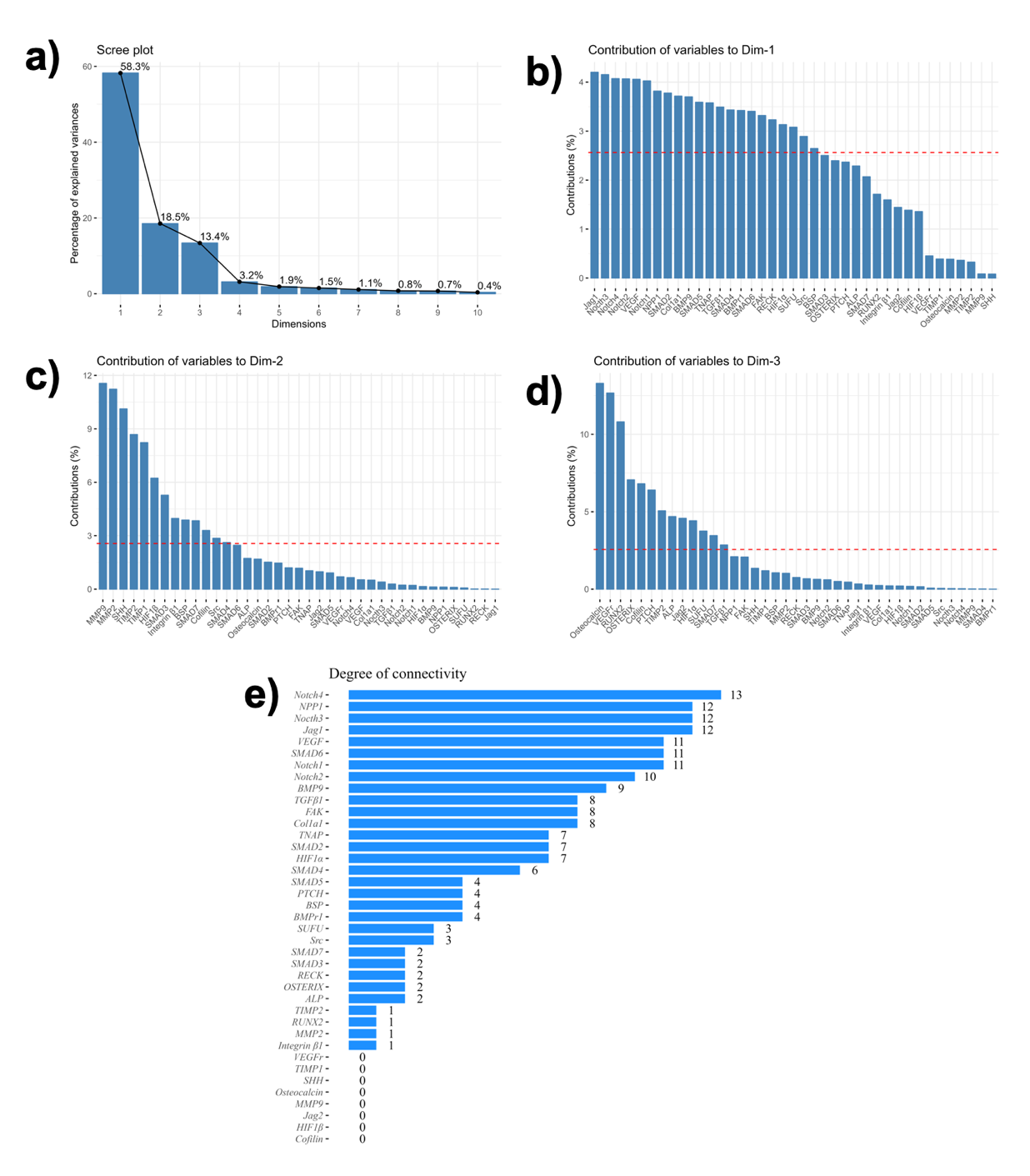


**Supplementary Figure 1.** *Principal Component Analysis (PCA) and Gene Network Visualization.* (a) Scree plot showing the percentage of variance explained by the first ten principal components. (b) Contribution of each gene to the first principal component (Dim1). (c) Contribution of each gene to the second principal component (Dim2). (d) Contribution of each gene to the third principal component (Dim3). Genes are arranged in descending order of contribution. (e) Bar plot displaying the degree centrality of each gene within the network. The network in Figure 8b, based on a correlation threshold of R² > 0.75, highlights a cluster of eleven genes (orange nodes) and isolates genes that do not form part of the primary network.
